# Shaped by the Industry: Female Fashion Models’ Perceptions of Aesthetic Labour, Body Governance and Disordered Eating

**DOI:** 10.3390/bs16060959

**Published:** 2026-06-10

**Authors:** Alison Fixsen, Reka Nagy, Magdalena Bailey

**Affiliations:** School of Social Sciences, University of Westminster, 115, New Cavendish St., London W1W 6UW, UK

**Keywords:** models, fashion industry, objectification, aesthetic labour, eating disorders, body surveillance

## Abstract

Female fashion models face intense bodily surveillance within a competitive and largely unregulated industry. While many young recruits endure the industry’s initial demands, less is known about how these formative experiences are later interpreted. Grounded in feminist and phenomenological perspectives, this article presents an inductive analysis of 18 semi-structured interviews with female models aged 25+, exploring entry into modelling, socialization into industry norms, and longer-term effects on body image, eating attitudes, and embodied practices. Five interconnected themes were identified: growing up “superfast”; learning to eat less; being treated as a product; boundary violations as a darker side of modelling; and building resilience within a callous industry. Early immersion in modelling culture shaped psychological development, with self-worth becoming closely tied to conformity with a narrowly defined thin ideal. Aesthetic labour extended beyond paid work to continuous bodily regulation and self-surveillance, producing a persistent tension between the lived, sensing body (Leib) and the body as an object for evaluation (Körper). Disordered eating emerged as a normalised employment strategy. While maturity could soften this process, participants described ongoing attempts to reconcile bodily needs with external judgements. The fashion industry thus emerges as a concentrated site of objectification, with enduring consequences for embodied wellbeing.

## 1. Introduction

Despite decades of feminist and body-positivity critique concerning narrow ideals of the “perfect” female body (Bordo, 2003; Gill & Scharff, 2011), mainstream media has continued to valorise an ultra-slim aesthetic (Fixsen, 2024). The fashion industry has been highly complicit in this: its relentless pursuit of youthfulness and thinness not only entrenches harmful norms but sustains ongoing concerns about its complicity in the production of disordered eating (Levine & Murnen, 2009). Central to this problem—and to this article—are the models themselves, many recruited at moments of profound psychological and physical vulnerability (Calogero et al., 2011; Tiggemann & Slater, 2014). While modelling promises glamour, status, and international mobility, these rewards mask a deeply unequal power structure in which agents dictate aesthetic requirements, and young models’ careers and sense of worth often depend on sustaining a BMI (Body Mass Index) that clinical professionals would condemn as dangerous. Research has repeatedly exposed the industry’s “toxic” underside, where restrictive eating, substance use, and other health risks are normalised as conditions of employability (Rodgers et al., 2020). While many young models endure the industry’s initial demands, far less is known about how they later interpret these formative experiences—particularly how, with greater maturity and hindsight, they understand the long-term health and psychological consequences of early and intense bodily scrutiny. This absence of critical retrospective accounts represents a notable gap in scholarship on modelling, embodiment, and disciplinarity.

Our article addresses this gap by interpreting the narratives of eighteen female models, tracing their entry routes into the industry, their socialisation within modelling culture, and the enduring impacts on body image, eating attitudes, and embodied practices. The analysis draws on phenomenological understandings of embodiment as sensitising concepts to explore how modelling work may shape embodied self-relations over time, including how bodily evaluation becomes internalised and expressed through everyday eating practices.

### 1.1. The Aesthetic Labour of Fashion Models

What Hochschild (1983) calls ‘emotional labour’ refers to managing feeling to create a publicly observable display that meets occupational expectations. ‘Aesthetic labour’ extends this to the commercial regulation of appearance, behavior, and self-presentation (Warhurst & Nickson, 2007). This effect is intensified in fashion modelling, as the body itself becomes the product (Entwistle & Mears, 2012). For models, the body is not merely present at work; it is the foremost “tool of the trade” (S. Holla, 2016), complicating the relationship between work, embodiment, and identity (Fixsen et al., 2023). The fashion sector is marked by structural contradictions, where exploitative production coexists with the glamor of high fashion (Arvidsson et al., 2010; Hoskins, 2014). Models, though not on manufacturing floors, face precarious employment, often lacking protections such as sick pay or parental leave (Hoskins, 2014). Within a largely unregulated market, female models are subject to strict bodily rules privileging youth, thinness, and precise measurements (S. Holla, 2016; Mears, 2010).

This constant scrutiny creates conditions in which mental health challenges are common (Fortune Super et al., 2023). Narrow beauty ideals (Mears & Finlay, 2005), existing alongside continual appraisal of body proportions, undermine emotional wellbeing and self-concept (Anderson et al., 2014), increasing models’ vulnerability to anxiety, depression, diminished self-worth (Kinderman et al., 2015; Meyer et al., 2007), and body-related nervousness (Carr & Mercer, 2017). This repeated external evaluation encourages an objectified relation to the body, where the model perceives themselves as reduced to their physical appearance. 

Unlike many forms of service work, models must remain continuously “industry ready,” extending labour beyond formal working hours (Mears, 2011). This leads to a state of ongoing bodily vigilance (Entwistle & Wissinger, 2006). Networking events further blur boundaries between work and personal life, intensifying the merging of body, identity, and labour (S. Holla, 2016). Aesthetic labour thus becomes totalising, embedded in continuous self-presentation and discipline. Despite these risks, mental health provision within agencies remains limited and psychological distress is often overlooked (Fortune Super et al., 2023).

### 1.2. Body Surveillance and Objectification

One way of approaching models’ bodies is through the phenomenological distinction between “Leib” (the lived, sensing body) and “Körper” (the corporeal, objectified body), (Husserl, 1989), wherein the body is both the site of lived experience and an aesthetic object shaped by representation. Husserl posits that the lived body is not merely a biological vessel but the primary site of perception, experience, and identity, through which individuals relate to the world. However, the experiential selfhood is fractured when the body becomes subject to institutional control, shifting from Leib to Körper. The tension between the body as medium of experience (Leib) and object within a system becomes particularly salient in the modelling industry where bodies are disciplined, stylised, and rendered visible as aesthetic objects, often at the expense of their lived, sensory, and affective dimensions.

From a feminist perspective, both agents and models’ experiences are situated within broad patriarchal structures that sustain narrow, gendered beauty ideals and position women as objects of the male gaze (Bordo, 2003; Fixsen, 2024; S. M. Holla, 2018). These ideologies have historically justified women’s subordination by constructing their bodies as fragile, malleable, and in need of discipline (Bordo, 2003; Grosz, 1994). From an early age, models are subjected to intense bodily surveillance within a competitive and often exploitative industry (Fixsen et al., 2023; Giotta, 2021). Frequently scouted young, they are expected to continuously reshape their bodies in response to shifting commercial demands (S. Holla, 2018), even amid natural physical development. This produces a tension between biological maturation and the industry’s preference for youthful thinness. In phenomenological terms, such conditions could be said to disrupt the unity of the Leib, as the body becomes something monitored, corrected, and disciplined, increasingly experienced as Körper rather than the immediate ground of perception.

As a form of aesthetic labour, modelling often requires the performance of a sexualised ideal (Warhurst & Nickson, 2009). Male-dominated roles—agents, designers, photographers—act as key arbiters of value (Crowley, 2021). Success depends not only on physical appearance but also on projecting confidence, approachability, and desirability, often through subtle flirtation (Entwistle & Mears, 2012). Trends such as “heroin chic” (Diaz, 2022) and “porn-chic” (Giotta, 2021) depict extreme thinness, infantilisation and sexualisation as acceptable and desirable (Sigdel, 2024). These expectations blur boundaries between performance and lived embodiment and intensifying emotional labour under precarious conditions, further entrenching the objectification of models’ bodies.

The modelling industry has been described as “brutal,” as it is marked by intense competition, coercive weight-loss practices, and precarious employment (Lewak & Ridley, 2017; Rodgers et al., 2020). Reports of sexual harassment (Gunawan et al., 2023), substance misuse, and mental health difficulties—including anxiety, depression, and suicidal ideation—are widespread (Dauxerre & Péronnet, 2017; Helmore, 2017). These conditions are compounded by entrenched, permissive behaviors around models and their bodies, including unwanted sexual attention and degrading treatment (Council of Europe, 2011; Crowley, 2021). That many models live away from family support from a young age increases their vulnerability to exploitation (Fixsen et al., 2023). Collectively, these dynamics shape not only careers but the phenomenological experience of embodiment, reinforcing objectification of the body as Körper.

Accounts from former models highlight the psychological consequences of these pressures (Dauxerre & Péronnet, 2017). Self-surveillance and body monitoring function as both outcomes and reinforcers of internalised ideals, contributing to body shame, diminished self-worth, and distress. Failure to meet shifting expectations generates inadequacy, self-hatred, and bodily alienation. Cycles of striving, failure, and self-punishment are intensified in environments rewarding extreme thinness and compliance, positioning the body as both capital and a site of control and discipline.

### 1.3. Restricted and Disordered Eating in the Modelling Industry

Eating disorders represent one of the most serious manifestations of the intersecting pressures on womens’ bodies. Defined as mental health conditions involving intense concern with body shape or weight, significant emotional distress, and maladaptive eating behaviors (American Psychiatric Association, 2022), eating disorders include anorexia nervosa, bulimia nervosa, binge-eating disorder, avoidant-restrictive food intake disorder, pica, and rumination disorder. Subthreshold eating disorders (SEDs) occupy a space between normative eating patterns and clinical diagnoses (Wacker & Dolbin-MacNab, 2020). Although often perceived as less severe, SEDs can be persistent and are associated with impaired functioning, comorbid mental health conditions, and elevated suicide risk (Flament et al., 2015).

While models may not exhibit significantly higher rates of clinically severe eating disorders than the general population, subclinical eating-disorder symptoms are particularly prevalent among models (Preti et al., 2008; Zancu & Enea, 2017; Bogár et al., 2022). Repeated messaging about weight and body size can negatively affect body image—understood as an individual’s thoughts and feelings about their body (Grogan, 2008)—and for models this can undermine their general psychological wellbeing (Anderson et al., 2014). For new and aspiring models, these pressures are compounded by frequent rejection, which demands substantial emotional resilience (Helmore, 2017). Expectations to lose weight—even among those already underweight (Dauxerre & Péronnet, 2017)—fuel a heightened drive for thinness (Swami & Szmigielska, 2013) and can lead to extreme weight-control practices (Lewak & Ridley, 2017). Reports indicate that some models resort to self-induced vomiting, stimulant use, intravenous drips, or the consumption of non-food items, practices that intensify both physical and psychological strain (Clements, 2013; Rodgers et al., 2020; Santonastaso et al., 2002). 

Studies suggest that certain structural factors actively encourage severely restricted and disordered eating behaviors in models (Fixsen et al., 2023; Rodgers et al., 2017, 2020). In a 2017 survey, over half of models had been threatened with job loss if they did not lose weight, prompting engagement in harmful practices such as meal skipping, illicit drug use, intravenous drips, or self-induced vomiting (Rodgers et al., 2017). Such behaviors have serious health implications, especially for very young models, including impaired growth, delayed puberty and disrupted menstruation (Neale et al., 2020), and the risk of skeletal complications (Donaldson & Gordon, 2015). Taken together, the literature indicates that the modelling industry does not merely reflect dominant beauty standards; it actively reproduces and intensifies them, creating conditions in which disordered eating behaviors can emerge and persist.

### 1.4. Rationale, Aims and Conceptual Framework

Our study explored the lived experiences of women working in the modelling industry, focusing on their perceptions of the psychological and physical costs of aesthetic labour for themselves and others. Drawing on retrospective accounts from current and former models, the study utilises hindsight to critically reflect on early entry into the industry. The research is informed by a feminist, constructionist perspective which views experiences of embodiment as shaped by social and cultural and institutional contexts. Participants’ accounts were considered in relation to broader industry regulations around regulation, appearance, performance and value.

The analysis was primarily data driven; however, Husserl’s (1989) distinction between Leib (lived body) and Körper (object body) was identified during analysis as a sensitising concept for interpreting tensions between embodied experience and external evaluation. The concept of the “sticky relationship” emerged at the final stage of analysis. The study aimed to contribute to understanding how industry practices are internalised and how these may relate body dissatisfaction and disordered eating, particularly when individuals enter the profession during formative developmental stages.

## 2. Materials and Methods

### 2.1. Study Design

A qualitative design was used, based on the secondary analysis of two closely aligned datasets comprising semi-structured interviews with professional fashion models. Although the original studies were conducted independently, they shared similar design, sampling, and data collection procedures, allowing them to be integrated into a single analytic corpus. The analysis was exploratory and interpretative, enabling close engagement with lived accounts, prioritising depth and richness of insight and giving “power to respondents” (Elliott & Timulak, 2005).

### 2.2. Participants and Recruitment

The dataset consisted of 18 semi-structured interviews conducted between 2019 and 2024. Participants were recruited using purposive sampling, with recruited independently through professional modelling networks rather than through a single institution, agency, or clinical centre. Participants were or had recently been working as models in commercial and/or high fashion.

### 2.3. Inclusion and Exclusion Criteria

Participants were included if they had professional experience of fashion modelling, were aged 24 years or older at the time of the interview; and had a minimum of two years’ experience in the industry. Participants were excluded if they reported current experience of an eating disorder, to minimise risk of distress.

### 2.4. Participant Characteristics

Participants’ ages ranged from 25 to 35 years. Available demographic information included nationality and age at entry into the modelling (Table 1). Modelling experience ranged from 5 to 15 years. Data on employment outside modelling were not consistently collected and are therefore not included.

### 2.5. Data Collection

Data were generated through one-to-one semi-structured interviews, conducted either face-to-face or via a secure online platform. Interviews lasted 30–60 min. Interview topics included: entry into modelling; industry norms and expectations; body image and aesthetic regulation; eating practices and health impacts; support systems and coping strategies; suggestions for improving wellbeing.

### 2.6. Ethical Considerations

Ethical approval was obtained from the University Research and Ethics Committee. Participants provided informed consent, were debriefed, and assured of confidentiality and anonymity. Given the sensitivity of body image and eating-related topics, interviews were conducted with care and avoided unnecessary probing into distressing experiences. Present age, BMI and other physical details were not collected due to ethical concerns.

### 2.7. Data Analysis

Data were analysed using inductive thematic analysis (Howitt, 2019). A constant comparative approach (Boeije, 2002) supported systematic comparison across interviews and categories. Investigator triangulation was used to enhance analytic rigour through iterative team discussion. Following Heaton’s (2004) suggestions for secondary analysis, a new research question was used for this study: how do women reflect retrospectively reflect on entering the modelling industry during formative, developmental stages and how they make sense of the embodied psychological and physical costs of aesthetic labour?

**Figure 1:**
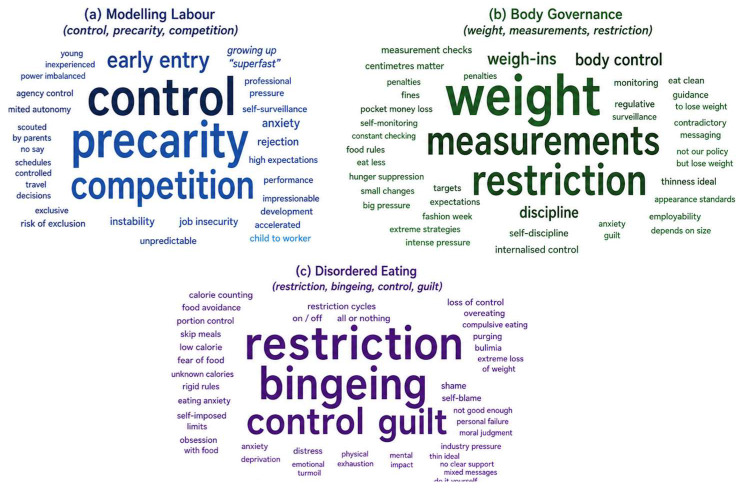
Word cloud clustering of participant concerns across three key domains: (**a**) modelling labour (control, precarity, competition, (**b**) body governance (weight, measurements, restriction) and (**c**) disordered eating (restriction, binging, guilt).

Analysis proceeded in three phases: Familiarisation where each author reviewed transcripts independently followed by coding and integration where datasets were merged and coded inductively. The first author conducted detailed coding using manual techniques and NVivo 14. A coding framework was constructed to collate codes and describe what each code encompassed. Codes were then examined collectively to consider how they might cluster into potential themes. This stage was iterative and reflexive; codes were grouped, refined, or set aside based on how meaningfully they captured participants’ experiences. A word cloud (Figure 1) was also created from the raw data to support the exploration of connections between codes and to identify overlaps and prominence of domains across both data sets. Theme development then took place with codes grouped into higher-order themes and refined through collaborative discussion.

### 2.8. Sample Size and Analytical Rationale

The sample size (n = 18) reflects the integration of the two methodologically aligned qualitative datasets. Given the focused population and shared experiential context, the dataset was sufficient to support thematic saturation and in-depth analysis, consistent with qualitative research principles prioritising analytic richness over statistical generalisation.

### 2.9. Overview of Data Collection Process

**Figure 2:**
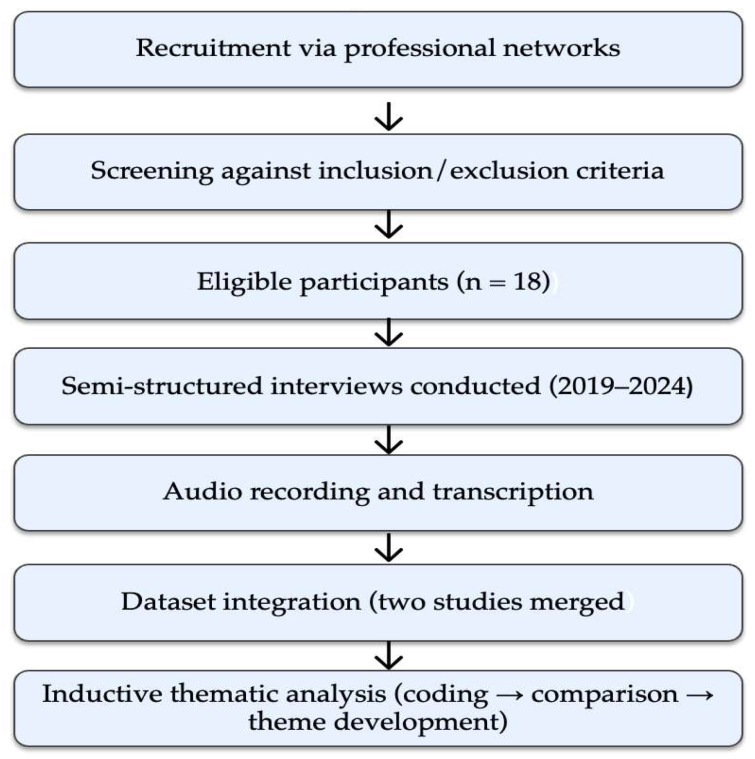
Flow diagram of qualitative data collection and analysis.

Figure 2 provides a flow diagram summarising participant recruitment, screening, interview procedures, and data integration involved in the collection and analysis of data.

### 2.10. Reflexivity

The research team brought complementary expertise to the study. The first author who oversaw both study design and questions, has extensive experience researching eating practices and body image across diverse populations. The second and third authors’ prior experience as fashion models, alongside training in psychology, facilitated access to participants through professional and social networks within the modelling industry, with some participants known through previous work or shared agencies. This insider position supported rapport and openness when discussing sensitive issues such as body image, eating practices, and industry pressures, while enabling attention to implicit norms and industry dynamics. However, this introduced potential challenges. Participants occasionally assumed shared understanding, resulting in less detailed accounts, while the authors’ prior experiences may have influenced data collection and interpretation. To mitigate this, reflexive practices were embedded throughout, including maintaining a reflexive journal, revisiting transcripts to ground interpretations in participants’ accounts, and regular discussions between authors. Moreover, the secondary analysis data allowed for a more nuanced perspective of findings. Collaborative theme development and investigator triangulation further supported analytic rigor.

### 2.11. Figure

**Figure 3:**
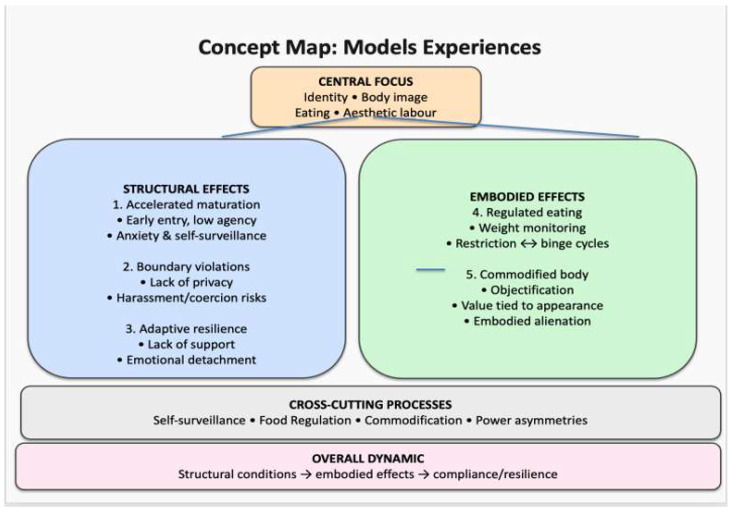
Concept map.

Figure 3 concept map illustrates how data relating to structural effects (e.g., recruitment practices, boundary violations, limited support) intersects with embodied experiences (e.g., eating regulation and body commodification), contributing to longer-term adaptive responses.

From these accounts, five interrelated themes were developed as presented below: (1) growing up superfast: entering modelling labour (2), body governance and regulatory practices (3), treated as a product: objectification and financial value (4) boundary violations: transgressing body and interpersonal limits (5) “fed very little”: restricted eating as normalised.

## 3. Findings

### 3.1. Theme 1: “Growing up Superfast”: Entering Modelling Labour

Participants reflected on entering the modelling industry at a highly formative stage, with most recruited before the age of 16. Caroline described being approached repeatedly at 14, noting that scouts often spoke directly to her parents rather than to her, effectively excluding her from decision making. Jules, recruited aged 13, recounted the disorientation of rapidly transitioning from a sheltered rural childhood into a metropolitan fashion environment, and pressure s to “grow up super-fast”:

“*I was scouted very young. It was on the school trip. Where I am from, nobody is really in the fashion industry, so I didn’t know anything about it. I had a very easy upbringing and nobody I knew was working in the fashion industry. So, I was quite intimidated, quite scared…*”

Being assessed on appearance at such a young age was described as destabilising, particularly in the absence of emotional maturity to contextualise rejection. As noted by Madelaine, many young recruits initially take rejection very personally: “*They’re not seeing it as a redirection to maybe another job, and [that] can really, really impact… your mental health.*” In her view, appropriate mentoring was essential in mitigating these early psychological impacts.

In the early stages, these pressures and anxieties could be accompanied by excitement and anticipation, with the modelling world offering exposure to a seemingly glamorous lifestyle. However, in Beth’s case, the initial boost in self-confidence was replaced by a hyper-awareness, with anxiety centred around appearance and evaluation:

“*Something happened between 18 and 21 when I was very anxious and very sort of… hyper aware of myself and my surroundings… I was also shaped in the way that I am constantly thinking about how I look and how that affect my overall value.*”

Despite an assemblance of independence, participants described highly structured and managed living conditions, with limited financial autonomy. As Caroline explained:

“*We were independent, but everything was taken care of… They would book you tickets, they would book your apartment, everything was fixed for you.*” She described this experience as “*just being in a bubble, it’s not real life.*”

Although refusal to cooperate was technically possible, it was often perceived as unrealistic due to fears that declining opportunities might damage one’s prospects. As Alice explained:

“*I didn’t personally feel very in control of my own career. My agent was in control of everything and kind of just told me what I was going to do… If you said no, it would affect all your future opportunities.*”

### 3.2. Theme 2: Body Governance and Regulatory Practices

This theme captures the core regulatory processes shaping participants’ experiences, including surveillance, measurement, discipline, and the gradual internalisation of these practices.

All participants described strict expectations regarding body size and appearance, with routine monitoring through weigh-ins, measurements, and informal feedback, establishing narrow parameters of what look was desirable. For those entering prior to full physical maturity this was particularly fraught. Normal developmental changes were often problematised; Nina described puberty as treated as deviation, while Olivia recalled being instructed to reduce her hip measurements without guidance:

“*Nobody talked about [menstruation] with me when I was like 15 in agency. Somebody was just measuring me and saying, ‘Oh, you need to lose four centimeters on your hips. Do it now. I don’t care how you do it, just do it.*”

Measurement regimes appeared to function as disciplinary mechanisms. Beth described feeling “*slapped in the face*” when her measurements exceeded industry thresholds; a moment that highlighted for her how these systems functioned as disciplinary tools. Lotti’s account of losing essential pocket money when measurements increased by even one centimetre illustrates how these practices operated not only as external sanctions but as technologies of self-regulation, encouraging constant monitoring and adjustment:

“*In China every week you had to go to the agency because they would check your weight and measurements… for some girls they wouldn’t give them pocket money if your measurements would be 1 cm bigger. And this money is basically for living.*”

(Lotti)

Such practices were described as operating both externally and internally. Over time, participants described becoming highly attentive to food intake, body size, and minor physical changes, with the threat of rejection reinforcing vigilance.

Periods such as fashion week intensified these pressures, sometimes resulting in extreme strategies to suppress hunger. Institutional messaging was experienced as contradictory, promoting “health” while encouraging weight loss:

“*I was given an ultimatum that I needed to get plastic surgery; otherwise, I couldn’t continue. […] So, I ended up getting plastic surgery at 15.*”

(Alice)

### 3.3. Theme 3: Feeling Treated as a Product: Objectification and Financial Value

Building on pressures identified in theme 2, participants described a distinct experience of objectification, in which their bodies were positioned as commodities within a market system. Many felt they were not recognised as “*more than just the face*,” reflecting a broader sense that their identities were overlooked. Participants reported feeling reduced to dehumanised roles, with metaphors such “*doll*,” “*hanger*,” or “*mannequin*,” highlighting reduction in personhood functional appearance. As Beth explained, being treated as a product in a market system felt an affront to her personal identity:

“*Others might look at you more like a tool—basically just something to show off their product. They want to get the most out of that tool, which is the model, and really push you to work, work, work because they’re paying for it.*”

Objectification was closely linked to profitability. Praise and recognition were contingent on booking frequency, creating a dynamic where interactions depended on productivity:

“*If you work a lot, you’re praised… but if you don’t work a lot then everybody just looks at you and it’s like, you know, the energy. […] It’s all shallow.*”

(Caroline)

Participants also described expectations of compliance, with little scope for expressing opinions. Mary characterised herself as “a piece of meat,” while Olivia described feeling like “a pawn in the game.” Jane reflected on how passivity was treated as part of the job:

“*But the reality is that it’s a real job, and then you’re treated like an object a lot of a time. Then people expect you to be passive and just not to have opinion on anything…*”

Experiences during fittings and long working hours contributed to a sense of bodily detachment. Olivia recalled standing for extended periods while garments were altered directly on her body, sometimes resulting in injury, alongside a sense of disconnection:

“*I remember that sometimes my body didn’t feel like mine. Um, for example, during fittings, I would stand for hours on high heels, sit or wait endlessly, or even have garments stitched directly onto my body with needles. Sometimes, those needles would pierce my skin because, uh, the designers weren’t careful. And at that time, I didn’t even feel it… or rather, I felt like I wasn’t even in my body.*”

Participants described being expected to appear poised even when experiencing discomfort or strain. Beth highlighted the ambiguity of these expectations:

“*They would give you these cues and I wouldn’t know what they meant. Like, what’s ‘being expensive’ supposed to mean? Should I go and buy Gucci clothes or what should I supposed to do? Or other things that they say, ‘your body isn’t that good, you’re not really a runway girl anymore’, which implied that I didn’t have a skinny body anymore.*”

(Beth)

Participants also described pressures linked to work assignments and travel, with limited transparency or control. Mary described being sent to different markets with little explanation or regard for her financial security: “*Basically, I felt like a piece of meat… whoever wanted me they just sent me there without a reason.*”

Participants felt agencies treated them as financial assets with a “*price tag*”, with their worth tied on with frequency of bookings and profitability. Madelaine described being seen as “*the price they’re paying for*,” while Charlotte noted that agencies’ financial interests shaped professional relationships. Olivia struggled to find the right term to describe this type of humiliation noting: “*I don’t want to use the word slave, but it’s just like you’re just… the player in the chess, a pawn, you’re just a pawn in the game.*”

These accounts illustrate how being treated as an aesthetic product—objectified, evaluated, and monetised—undermined models’ agency and sense of self, contributing to uncertainty about their value and position within the industry.

### 3.4. Theme 4: Boundary Violations: Transgressing Bodily and Interpersonal Limits

In addition to feeling themselves evaluated as products, participants described experiences that involved direct violations of physical and interpersonal boundaries, including lack of privacy, unwanted contact, and inappropriate professional conduct. These included lack of privacy when changing, unwanted physical contact, and explicit commentary on intimate aspects of the body. Participants described early normalisation of such practices that were later recognised as problematic, including undressing on front of others, and exposure to explicit commentaries about their bodies. Madeline recalled frequent remarks about intimate body parts:

“*I heard someone saying to lose weight and then your ass isn’t big enough and your boobs are too big or too small, you know… they were even commenting on my teeth, and my teeth are not so straight.*”

(Madelaine)

According to Caroline, boundary violations began early in her career and felt inappropriate in retrospect:

“*…and this is one of the things that I’m really used to… from a young age it’s just very normal to undress in front of a lot of people, which is also a bit strange really, you know. […] You are still a child; you’re still underage. It’s not okay, actually…*”

Boundary issues also involved unwanted physical contact. Olivia recalled: “…*when changing clothes, someone would come to adjust a garment and touch me near my chest or vagina. There were no clear boundaries.*”

Two participants raised broader concerns about inappropriate professional conduct. Lily recalled being invited to events by promoters who were later revealed to be financially motivated, blurring the line between modelling and escorting, particularly when arranged by agencies. Charlotte described older agents and photographers pursuing romantic relationships with young models, raising concerns about exploitation.

Participants were aware that sexual harassment occurred in the industry and emphasised the vulnerability of younger models. While most mentioned this briefly, Caroline shared a more intimate experience involving older photographers who offered her alcohol and attempted to initiate sexual contact:

“*I did have, for example, photographers trying to reach out and then trying to have sexual relationships with you, but you’re very young and the photographers’ maybe in their thirties already… offering you alcohol after the shoot or taking shots like that.*”

The knowledge that in some “professional” situations, sexual advances might take place, created anticipatory anxieties for some participants. Caroline explained how she was reluctant to fly to New York for a one-day shoot because; “*the guy also had a bad reputation with like, kind of sexual assault… or being a bad guy*.” Yet, for many young models, submission to the demands of the manager and client might seem like the only way to retain one’s job:

“*You have this choice, either you submit, or you strengthen yourself and become your own individual. Um, but I think in most cases, it is submission.*”

(Olivia)

Significantly, these accounts suggest that bounty violations were not experienced is isolated incidents but were embedded within wider work conditions in which consent, autonomy, and safeguarding were weak or absent.

### 3.5. Theme 5: “Fed Very Little”: Restricted Eating as Normalised

Building on the regulatory processes outlined in Theme 2, restricted eating emerged as a distinctive and significant aspect modelling work. Although individuals with diagnosed eating disorders were excluded, many described patterns of eating that became increasingly controlled or disordered over time.

These patterns often developed gradually. Learning to eat less was described as a subtle, incremental process shaped by the demands of the modelling environment. Lily recalled long working days during which models were “*fed very little*,” or sometimes not at all, suggesting that eating was frequently incompatible with the rhythms and expectations of the job. Over time, such external pressures became internalised. As Jules reflected, weight loss was positively reinforced regardless of how it was achieved:

“*I got down with my measurements, and I was congratulated… looking my slimmest and hearing that I have never looked better. It just felt wrong.*”

(Jules)

For some, initially moderate dietary changes became increasingly rigid. Jane described how avoiding meat evolved into constant calorie counting, documenting of all food intake, and avoidance of social eating contexts:

“*So, I started not eating meat… I started counting calories. I was writing down everything I ate during the day… I couldn’t even eat salad in a restaurant because I didn’t know the exact amount of oil in it… If I got less than 1200 calories that felt amazing.*”

For some, restrictive patterns led to cycles of fasting and binging. Lotti, who once lost 10 kg in a month, later recalled, “*stuffing [her] face*” with granola, explaining that “*if you restrict something that much then you just can’t handle it anymore.*” Caroline linked her years of battling with bulimia directly to modelling: “*If I never started modelling, I would never have these issues to that extent.*”

Importantly, participants consistently located these changes within the broader modelling environment rather than individual predisposition. While agencies occasionally discouraged extreme or visibly “unhealthy” behaviours, this was experienced as inconsistent and unsupported. Institutional messaging about self-care was described as contradictory, with agents paying lip service to mental wellbeing and healthy eating yet simultaneously circulating guidance encouraging weight loss:

“*They would send out little leaflets or emails… with advice on how to lose weight or eat less but stay healthy. But it was a bit of a bullshit because… it wasn’t really clear or evidence based.*”

In the absence of clear guidance, participants were left to manage expectations independently. As Madelaine noted, “*There is not even a single coach. There is no one you can talk to about these kinds of problems.*”

Taken together, these accounts indicate that disordered eating practices in this context are not easily understood as individual pathology. Rather, they emerge within a system of regulatory pressures embedded in modelling labour, in which bodily discipline is both normalised and tacitly rewarded.

## 4. Discussion

Our study examined fashion models’ pathways into the industry, their experiences of modelling culture, and perceived impacts on body image and eating attitudes and behaviours. Five distinct but interrelated themes were identified: growing up superfast: entering modelling labour; body governance and regulatory practices; treated as a product: objectification and financial value; boundary violations: transgressing body and interpersonal limits; and “fed very little”: restricted eating as normalised. Drawing on a phenomenological perspective, participants described modelling as a context in which visibility, bodily evaluation, and appearance-based expectations became particularly salient. Across retrospective accounts, heightened attention to appearance and bodily monitoring emerged as central features of everyday experience, becoming embedded over time in routine practices and ways of relating to the body. Participants’ accounts include trajectories in which externally imposed expectations of thinness transitioned into increasingly structured and restrictive eating practices over time. Practices such as calorie counting, food avoidance, and cycles of restriction and bingeing were commonly described. The conviction that guidance was absent or lacking in credibility reinforced the sense that institutional outcomes (notably thinness) were prioritised over how they were achieved.

Taken together, the findings position modelling as a setting in which body management, professional expectations, and self-evaluation become closely intertwined. The rest of the discussion focuses on three key areas: aesthetic labour and objectification, boundary erosion within the modelling environment, and the normalisation of restrictive eating practices, with a “sticky relationship” developing between modelling culture and restricted and disordered eating.

### 4.1. Aesthetic Labour and Objectification

Previous research conceptualises fashion modelling as involving both affective and aesthetic labour (Fixsen et al., 2023; Giotta, 2021), requiring emotional performance alongside appearance regulation (Warhurst & Nickson, 2007). Many participants characterised their entry into modelling as externally initiated through scouting, echoing Entwistle and Mears’s (2012) description of models as “discovered” rather than self-directed. Participants described learning to project emotional states and attune to the demands of shoots and castings, consistent with prior work on modelling labour (Wissinger, 2007). Their accounts also suggested that the body could come to be experienced less through lived immediacy and more through external evaluation, resonating with Orbach’s (2009) notion of the body as “an aspiration always needing to be achieved” (p. 145).

Consistent with previous studies (Giotta, 2021; S. M. Holla, 2018), participants described repeated experiences of objectification during castings, fittings, and measurements, where bodies were framed as commodities, “products,” “tools,” or “mannequins.” Feedback often focused on isolated body parts, contributing to participants’ experiences of bodily fragmentation and heightened awareness of appearance. Participants also described pressure to maintain very low measurements, while industry language such as “in shape” or “show ready” was sometimes perceived as obscuring the strain associated with these expectations. Phenomenologically, these experiences may reflect a shift in how participants related to and experienced their bodies under objectifying conditions—from the body as immediately experienced (Leib) toward a sense of the body as an object of external scrutiny and evaluation (Körper).

Participants further described modelling as a highly competitive and unpredictable environment characterised by limited autonomy and inconsistent support. For some, entering modelling at a young age was retrospectively understood as involving unequal power dynamics and accelerated professional expectations (Paccione, 2016). These accounts are broadly consistent with previous research depicting modelling as precarious and weakly regulated work (Maskell, 2019; Newby et al., 2021). Several participants reflected on navigating adult work environments, international travel, and financial pressures during adolescence while feeling developmentally underprepared. Consistent with earlier findings, mental health support was generally perceived as exceptional rather than routine (Newby et al., 2021; Rodgers et al., 2017). Participants commonly described feeling responsible for independently managing nutrition, fitness, and wellbeing while also meeting strict appearance expectations. Younger models were often perceived as particularly vulnerable because of limited resources, experience, and bargaining power.

### 4.2. Body Monitoring and Boundary Erosion

Whilst girls and young women more broadly are socialised to prioritise appearance (Bordo, 2003; Grogan, 2008; Mills et al., 2017), participants described modelling-specific body governance as narrower, more pervasive, and more difficult to disengage from (Entwistle & Wissinger, 2006; Fixsen et al., 2023; Mears & Finlay, 2005; Rodgers et al., 2017). Over time, evaluative standards were often described as becoming increasingly internalised, with some participants finding it difficult to distinguish between professional feedback and broader perceptions of self-worth.

Consistent with first-hand accounts in the literature (Dauxerre & Péronnet, 2017), body humiliation and blunt managerial judgements were also experienced as routine by some participants. Several participants perceived the industry as structured around unequal power relations in which approval from agents, clients, and casting professionals was central to career progression, echoing Crowley’s (2021) discussion of “kingmakers.” Boundary violations were also frequently described as normalised aspects of work environments (Council of Europe, 2011; Crowley, 2021). Participants recounted expectations to undress in front of others, experiences of invasive touching, and exposure to situations they interpreted as sexually exploitative. Safeguards such as age protections and health standards were often perceived as inconsistently implemented (Fortune Super et al., 2023; Rodgers et al., 2017; Treasure et al., 2008). These experiences were commonly associated with feelings of anxiety, insecurity, and heightened awareness of the body.

### 4.3. Modelling Culture and Restrictive Eating: A “Sticky” Relationship

While population studies suggest that models do not exhibit significantly higher rates of clinical eating disorders than the general population, subclinical eating disorder symptoms appear relatively common in models (Preti et al., 2008; Zancu & Enea, 2017; Bogár et al., 2022). In the present study, participants frequently depicted restrictive and disordered eating practices as emerging or intensifying within the context of modelling work. Rather than being described as pre-existing, these practices were often retrospectively understood as developing over time through what we refer to here as a “sticky” relationship with food and restriction. This “stickiness” reflects the way eating practices can become bound up with everyday routines and professional demands, making them less readily identifiable as problematic within the context of modelling. For instance, many participants described how eating patterns were routinely organised around career concerns such as castings, shows and measurement sessions, rather than bodily cues such as hunger or satiety. Smaller portions, calorie counting, and avoidance of social eating were often framed as professionally necessary responses to perceived industry expectations surrounding thinness and inflexible rules around body dimensions. In this sense, restrictive eating was not described as a discrete behaviour, but as intertwined with decisions about work, appearance, and self-presentation. Consequently, models could fail to recognise points where eating practices shifted from occupational management into more distressing or long-lasting patterns such as binge eating and bulimia.

This interpretation aligns with broader work suggesting that restrictive eating can become embedded within habitual patterns of self-regulation (Bordo, 2003; Izydorczyk et al., 2020). Sociocultural perspectives further highlight the role of wider media and cultural influences in shaping body ideals and eating behaviours (Bogár & Túry, 2024 Lewak & Ridley, 2017; Izydorczyk et al., 2020; Tiggemann & Slater, 2014). Participants also referenced the increasing visibility of weight-loss medications, which were perceived as intensifying pressures towards thinness and optimisation. Regulatory developments, such as the UK Advertising Standards Authority’s rulings on “unhealthily thin” models (Advertising Standards Authority, 2025; Wright, 2025), indicate continuing tensions between commercial and public health concerns, which have yet to be with resolved.

Taken together, participants’ accounts suggest that the modelling environment was experienced as a context in which visibility, appearance regulation, and bodily evaluation were intensified. Early immersion in these conditions was retrospectively seen as contributing to heightened awareness of the body and appearance within everyday life. The findings therefore contribute to understanding how young women working in aesthetic labour contexts describe navigating pressures surrounding embodiment, visibility, and employability.

## 5. Implications for Intervention

Participants’ accounts of early body scrutiny, limited autonomy, and perceived exploitation highlight the potential value of strengthening labour protections and safeguarding practices within modelling contexts. While causal claims cannot be inferred from the present study, our findings strongly support existing recommendations to strengthen ethical standards, improve safeguarding policies, and increase access to psychological and nutritional support within the industry. Potential measures may include clearer minimum age requirements, independent welfare oversight, greater parental or guardian involvement for younger models, and structured industry inductions addressing rights, expectations, and support pathways.

Participants’ narratives also underscore the importance of recognising models as workers with rights rather than solely as aesthetic commodities. Improved transparency in agency practices, clearer expectations regarding privacy and physical contact during castings and shoots, and accessible reporting mechanisms may help address some of the concerns raised. Although several participants described developing resilience, this was often framed as an adaptive response to challenging environments rather than evidence of sufficient support. Consequently, interventions may benefit from focusing not only on individual coping strategies but also on broader organisational and cultural practices that participants perceived as shaping their experiences.

## 6. Limitations

As a qualitative study based on retrospective accounts from a relatively small purposive sample, the findings are not intended to be statistically generalisable to all fashion models. The study was also limited to participants who currently had no diagnosis of an eating disorder due to ethical restrictions. Participants, while representing a wide range of nationalities, were predominantly white-presenting women working within particular sectors of the fashion industry, and these experiences may differ across ethnic groups, body categories, genders, commercial sectors, and cultural contexts. The findings instead offer an in-depth exploration of how aesthetic labour and body regulation were experienced and interpreted within this specific sample. Future research should further examine how intersecting inequalities shape vulnerability, agency, and resilience within modelling and related forms of aesthetic labour. Longitudinal and mixed-methods research may also help clarify how industry experiences interact with broader sociocultural influences over time. At the same time, we trust that the present study offers noteworthy insights into how participants perceived the relationship between modelling culture, body regulation, and eating practices, contributing to broader discussions surrounding embodiment, labour, and gendered appearance norms. Our concept of the “sticky relationship” may also be applicable in other social and occupational contexts with aesthetic and weight dimensions.

## Figures and Tables

**Table 1:** Participants (pseudonyms, nationality, recruitment age).

Pseudonym	Nationality	Recruitment Age
Jules	British	13
Alice	American	14
Caroline	Belgian	14
Charlotte	Hungarian	14
Beth	Danish	14
Lotti	Russian	14
Olivia	Latvian	14
Nina	British	14
Emma	Hungarian	15
Jane	British	15
Mary	Ukrainian	15
Naomi	Polish	16
Anna	Polish	16
Karen	Polish	17
Alison	Australian	18
Lily	Hungarian	20
Madelaine	Slovenian	20
Rosie	Russian	21

## Data Availability

Further anonymized data is available on request.
